# In-Vivo Vibroacoustic Surveillance of Trees in the Context of the IoT

**DOI:** 10.3390/s19061366

**Published:** 2019-03-19

**Authors:** Ilyas Potamitis, Iraklis Rigakis, Nicolaos-Alexandros Tatlas, Stelios Potirakis

**Affiliations:** 1 Department of Music Technology & Acoustics, Technological Educational Institute of Crete, 71410 Heraklion, Greece; 2 Department of Electrical and Electronics Engineering, University of West Attica, 12241 Athens, Greece; i.rigakis@uniwa.gr (I.R.); ntatlas@uniwa.gr (N.-A.T.); spoti@uniwa.gr (S.P.)

**Keywords:** *Xylotrechus chinensis*, *Rhynchophorus ferrugineus*, precision agriculture, IoT

## Abstract

This work introduces a device for long term systematic monitoring of trees against borers. A widely applied way to detect wood-boring insects is to insert a piezoelectric probe with an uncoated waveguide in the tree trunk and listen for locomotion or feeding sounds through headphones. This approach has several shortcomings: (a) frequent manual inspection of trees is costly and impractical to scale to hundreds or thousands of trees, (b) the larvae could be present but inactive during the inspection time and, (c) when the trees are in urban environments the background noise can be significant and can mask the feeble sounds of wood-boring insects even with the use of specialized headphones. We introduce a remotely controlled device that records and wirelessly transmits on a scheduled basis short recordings of the internal vibrations of a tree to a server. The user can listen remotely or process the recording automatically to infer the infestation state of the tree with wood-boring insects that feed or move inside the tree. When integrated within the IoT framework this device can scale up to automatically monitoring the trees of the entire city. The proposed approach led to detection results in field trials of the pests *Xylotrechus chinensis* (Chevrolat) (Cerambycidae) and *Rhynchophorus ferrugineus* Olivier (Coleoptera: Curculionidae).

## 1. Introduction

Trees play a vital role in our planet and affect the life cycle of all living creatures. In the special case of urban trees, they provide cultural and aesthetic benefits beyond economic ones. Due to their importance, a range of sensors are currently available for measuring several vital parameters of trees related to health, growth and possible forest damage [1,2]. Yet, the penetration of technology in forest and urban trees management has been slower than in agriculture. Beyond the recording of several parameters that are correlated with tree health such as temperature, humidity, barometric data and precipitation there are several others less known to the generally knowledgeable audience. Dendrometers measure the diameter of trees and cross correlate this parameter with tree growth [3]. Accelerometers attached to the trees can provide long-term data series of tree sway, which is used to infer tree properties such as mass, elasticity, canopy precipitation and interception [4]. Sonic tomography is based on attaching several accelerometers to the trunk to assess the internal decay and cavities in living trees, and by measuring variation in the speed of sound across the trunk to determine patterns of wood integrity [5]. Another apparatus is the resistrograph that drives a drilling needle into the wood under constant drive [6]. The drilling resistance is measured at real time as the drill bit passes through the wood layers and reveals the internal decay [7].

Wood-boring insects such as termites, weevils and certain beetles can directly affect the health and growth of specific tree species. There is a wide bibliography on piezoelectric sensors, microphones, accelerometers, laser vibrometry and optical methods [1,8,9] used to detect locomotion and feeding sound of larvae or adult pests inside the tree trunk. One should note that all these methods are currently applied manually. In-situ manual application is the basic difference with our remote approach. The difficulty in detecting wood-boring pests lies in the fact that they breed inside the tree, and therefore, the tree itself becomes the host that provides a physical coverage. These pests inflict detrimental effects on the health of the tree and in many cases, such as *X. chinensis* for mulberries [10] and *R. ferrugineus* (also known as red palm weevil) for palms, they attack and kill living trees at high rates [11]. The signs of infestation are usually evident when it is too late for saving the tree and by then the adult pests have escaped from the tree trunk at large numbers to infest other trees.

Global trade and climate change facilitate the establishment of pest populations outside their native ranges. Larvae of invasive species breed in wooden pallets and are transported through ports, handling facilities or truck roads and, therefore, one after the other, species known to be ‘exotic pests’ appear in Europe [12] and US in places that are not biologically adapted to regulate their multiplication. Since identification of wood-boring larval insects is important for pest risk analysis and management, phytosanitary measures are commonly applied in points of entry such as commercial harbors and airports. Manual inspection of commodities by inserting a piezoelectric probe in the tree trunk to listen for potential internal audio activity due to feeding and locomotion (i.e., passive acoustic detection) is a widely accepted method [13,14,15,16] (listen also to Recording S1 in the Supplementary Materials). This approach has some distinct benefits, for example it is portable, has lower cost that competitive methods, can be operated with batteries, requires minor training and there are commercial products available [17]. It also comes with several practical shortcomings:

(a) Field visits and frequent manual inspection of trees and plants is costly and impractical to scale to large numbers,
(b) The human observer has limited time to inspect a single tree and the larvae could be present but inactive during the inspection time,
(c) When the trees are in urban environments the background noise can be significant and can cover the feeble sounds of wood-boring insects, even if the human observer uses specialized headphones. Moreover, the movement of tree branches or, in the case of palms, of the fibers of the trees due to the wind, produces interference sounds that resemble feeding sounds and can result in false alarms. A false alarm in this case entails a high cost, as the tree must be either treated and re-examined or removed and destroyed.
(d) Manual operation can be inconvenient and stressful, as observers needs to perform inspections in very noisy environments (e.g., inside cargo ships, on trees near high traffic), are exposed to weather conditions, often climb on ladders to reach the stems of very tall trees such as palm trees etc.). While being there the user must try not to make noise by himself, by movement of cables, handling of the probe, or even breathing, as the bouncing cables and cable headphones are sensitive to impact and create noise that obstructs the acoustical observation.

This work is a special case in our previous research activity [18,19,20], among other contributions [21,22]; it is our long lasting goal to establish automated insect surveillance at global scale for insects of economic importance. This work is about a novel accelerometer-based sensor that transmits short vibration clips stemming from an internal part of the tree to a remote server (e.g., 10 s of recording every hour). The server allows the user to have access to the audio recordings in a flexible platform with additional analysis tools for mapping, management and inventorying. The transmitted clips can be examined manually by hearing and visualization of their spectrogram, or automatically in order to assess the infestation status of the tree. Time scheduling is handled by the server, which communicates remotely with all deployed devices. Therefore, one can schedule recording sessions that would be inconvenient for manual practices (e.g., inspections during the night) and can be taken when background and/or traffic noise is low. Scheduling and automatic reporting can lead to a decision about the infestation state of the tree based on accumulating observations over a larger time span than the usual manual practice e.g., by examining the recordings of a week and accumulating experience before the final decision. 

**Figure 1:**
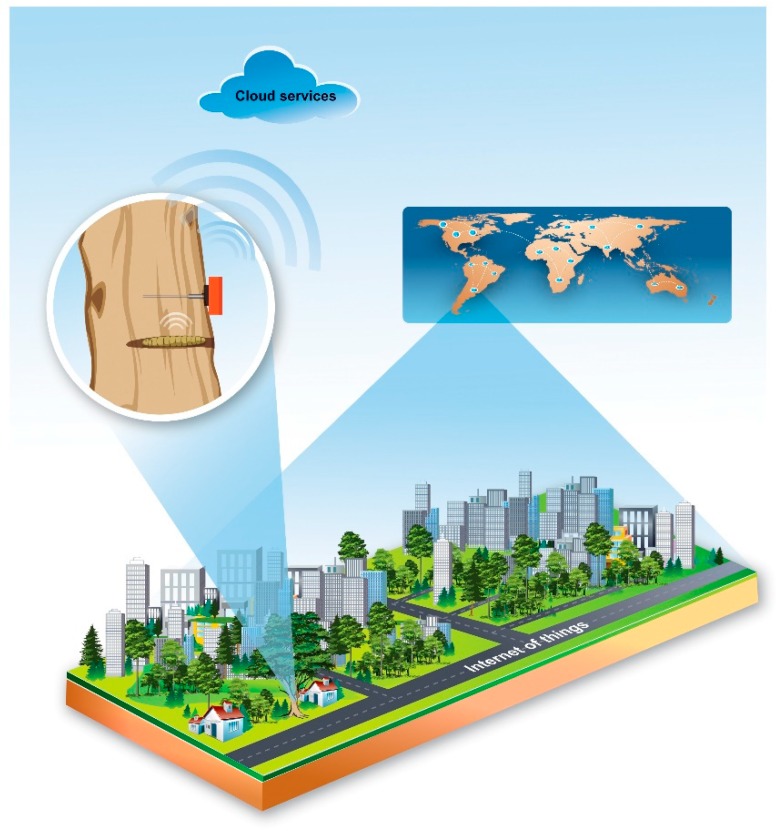
The concept of in-vivo acoustic surveillance of trees in forests and urban environments. Trees are probed for real-time monitoring against wood-boring insects. The device emits recordings of vibrations stemming from inside the tree trunk due to feeding or moving sounds of larvae. The IoT expands the remote monitoring application from a few trees to regional, national or global networks.

The manual visits are reduced to a single one, as the device is cost-effective (about 160 Euros as per 22 December 2018) and can be placed permanently on the tree (see Figure 1). If the observers hear, on time to the feeding sounds of the larva they can apply treatment using systemic insecticides or remove the tree to prevent spread of infestation and the after treatment monitoring cost related to the assessment of the efficacy of the treatment is minimized. The design of the device is low-power and with a small panel attached to its back surface can be power sufficient for many months. In this work, we apply the General Packet Radio Service (GPRS) functionality of the mobile network. Alternatively, the device can apply some basic feature extraction based on the RMS level, counts of impulses, impulses rate and then use the Long Range Wide Area Network (LoRa/WAN) communication protocol. LoRa/WAN can reduce significantly the communication cost and allow the application to be applied at large scales (hundreds to thousands of nodes) in the context of smart cities that already support the Internet of Things (IoT) infrastructure. 

The paper is organized as follows: first the methodology to retrieve the vibration signals and their frequency content is described in the ‘Materials and Methods’ section. Then, we present experimental results based on recordings of different species of insects one of them never reported in the literature. Finally, we discuss the possibilities of different applications of the device on the basis of our experimental results.

## 2. Materials and Methods 

**Figure 2:**
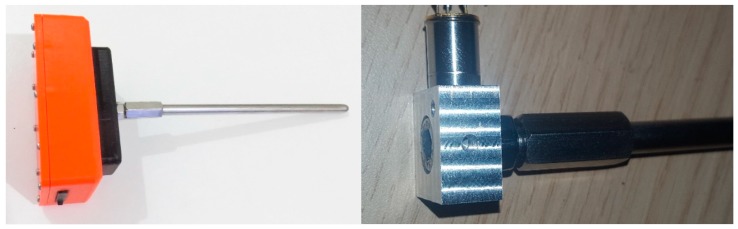
(**left**) The vibrations recorder: a rugged container for outdoor deployment contains the electronics including GPS and GPRS, (**right**). Detail of the metal waveguide fastened to the input of the accelerometer. The tree trunk is first drilled using a bit matching the size of the probe and the waveguide is subsequently inserted and fastened in the hole.

The device has 9 × 5 × 4.5 cm dimensions and is composed of: 

(a) a 16 cm long waveguide, (b) an accelerometer and (c) an electronic board that transforms recorded vibrations into lineout audio signals that are stored, compressed and wirelessly transmitted (see Figure 2). The waveguide is just a stainless steel bar, but large nails, bolts and drill bits can serve the same function, that is act as a sound coupler between the wood and the sensor probe. 

Drill bits can be conveniently adjusted to a drill bit adapter that, in turn, is fastened onto the accelerometer. Different waveguides apply to different user scenarios e.g., wooden columns, decks, porch tongues, ceilings, roots of plants. 

The waveguide is attached to an 805Μ1-0020 accelerometer (TE Connectivity Ltd, Rheinstrasse 20 Ch-8200 Schaffhausen, Switzerland), that is based on a stable piezo-ceramic crystal with low power electronics. We chose this accelerometer mainly for its frequency response (i.e., 1–8 kHz) where feeding impulsive sounds lie [8,16] and its sensitivity at 100 mV/g. However, besides these two criteria, we have not optimized the accelerometer selection and other choices may be applicable. The microprocessor carrying out all processing and communication with the submodules is the STM32L476RG (ST Microelectronics, Geneva, Switzerland). The STM32L476RG device is an ultra-low-power microcontroller based on the high-performance Arm Cortex-M4 32-bit RISC core operating at a frequency of up to 80 MHz. The device carries a GPS and a 4G-LTE SIM7600 (SIMCOM Shanghai, China), modem to transmit the recordings and the device’s position. 

**Figure 3:**
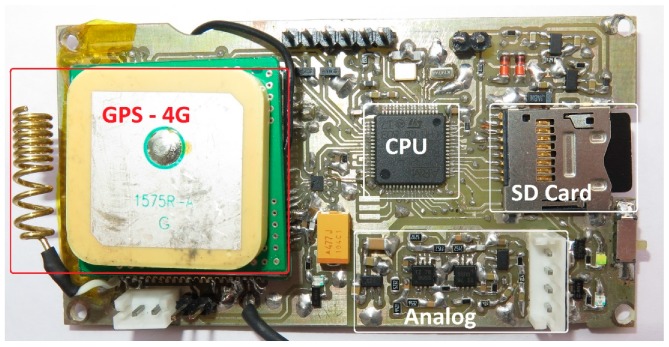
The recorder prototype. We circle and annotate the main components of the recorder. ‘Analogue’ denotes analogue amplification and low pass filtering using the micro-power operational amplifiers OPA313 (Texas Instruments, Dallas, TX, USA).

**Figure 4:**
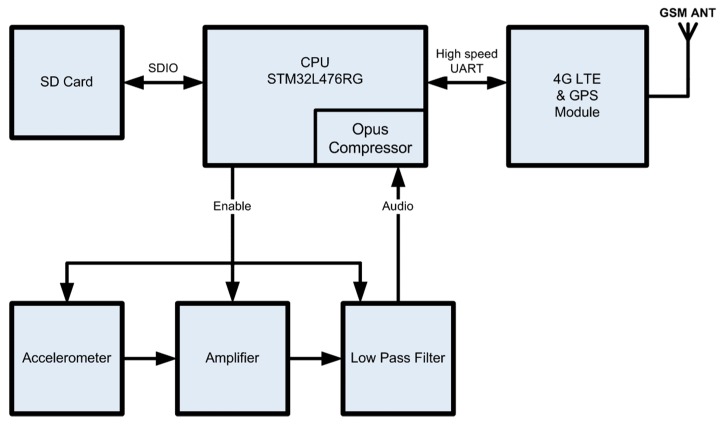
Block diagram of the vibrations recorder. The system is controlled by an STM32L476RG ARM CPU of ST. The 805M1-0020-01 accelerometer picks up the vibrations, that are amplified using the OPA313 and turns them into a line-out signal that is compressed, stored to the SD card and transmitted through the 4G module.

Prior to transmission, the waveforms are compressed using the Opus open source lossy audio coding format (http://opus-codec.org/). We show an internal picture of the completed device in Figure 3. The system’s sub-components are depicted in Figure 4. In the version of the device we introduce in this work, the circuit is constantly in sleep mode, wakes up on a predefined time schedule that is configurable through the reporting server and takes a recording before going to sleep again. 

A detailed power consumption calculation for a one user-case scenario can be found in the Appendix A.

There is also the possibility to run a constantly looping program that processes data captured by the sensor and records only when triggered by an audio event having power exceeding a threshold. The recording is stored in the SD card, and the time stamp is passed to the filename. All recordings are compressed using the open source Opus compressor prior to sending them over the communication channel. The bit rate is 24 KBPS at a sampling frequency of 8 kHz and effects a compression rate 5 to 1, meaning a wav file of 320 KB is compressed to 63 KB. We use global SIM cards, therefore any tree can be tracked from anywhere in the world. 

The board is programmed in C/C++. The output of accelerometer is amplified and filtered. The analog filter output is converted to digital words in 16-bit resolution at 8 kHz sampling rate using the internal ADC of the microcontroller. The sampling frequency, record duration and other initialization parameters are read once from the SD card during powering-on and are configurable through the server. The software is written in C language using the IAR Embedded workbench. The programming of the flash memory was carried out using the ST-Link V2 programmer. The code initialization was done using the STM32CubeMX of ST. For programming the peripheral sub-components such as the SD and ADC we made use of the STM32 HAL Drivers. The control signals and data transfers were implemented using the DMA controller of STM32L476. The recorded files are compressed using opus encoder and uploaded to a web page using a 4G-LTE module.

**Figure 5:**
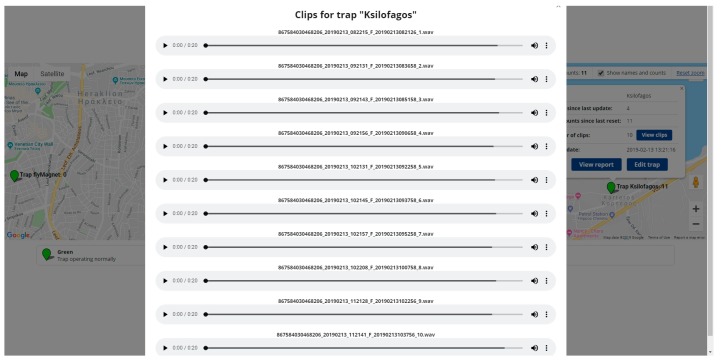
The reporting server manages recordings clips, tracks geospatial data, handles data inventorying and communication with all deployed sensors.

The server is described in [18] and manages the data collection and time-stamping, GPS registration and storage process of submitted audio recordings (see Figure 5). Moreover, the administrators can manage and customize the data fields and collection process as well as communicating commands to the sensor nodes. The 4G modem of the trap or the gateway in the case of LORA/WAN, once connected to the mobile provider, are capable of having internet connectivity. The trap makes a TCP/IP connection to the webserver of the backend, via a POST request, and uploads the recorded audio files. At this point, the trap inserts its data as parameters for the page that it wants to access. Once the HTTP request reaches the web server, the latter receives the data from the request of the trap (via the appropriate code, written in PHP) and logs the information in the database. The web application consists of two parts: The backend that manages the information and the database in the server and a frontend that visualizes the information at the browser of the user and the interface with the user. The site is based on a web hosting provider running Apache, with PHP 5.5 support. The database is setup using MySQL, which is open source. 

The backend is written in Laravel 5.1 PHP 5.5 and the frontend is written in HTML 5, making use of the Angularjs Javascript and JQuery javascript Framework. The data follow the JSON formalization. The maps are currently provided via the Google Maps API.

## 3. Results

**Figure 6:**
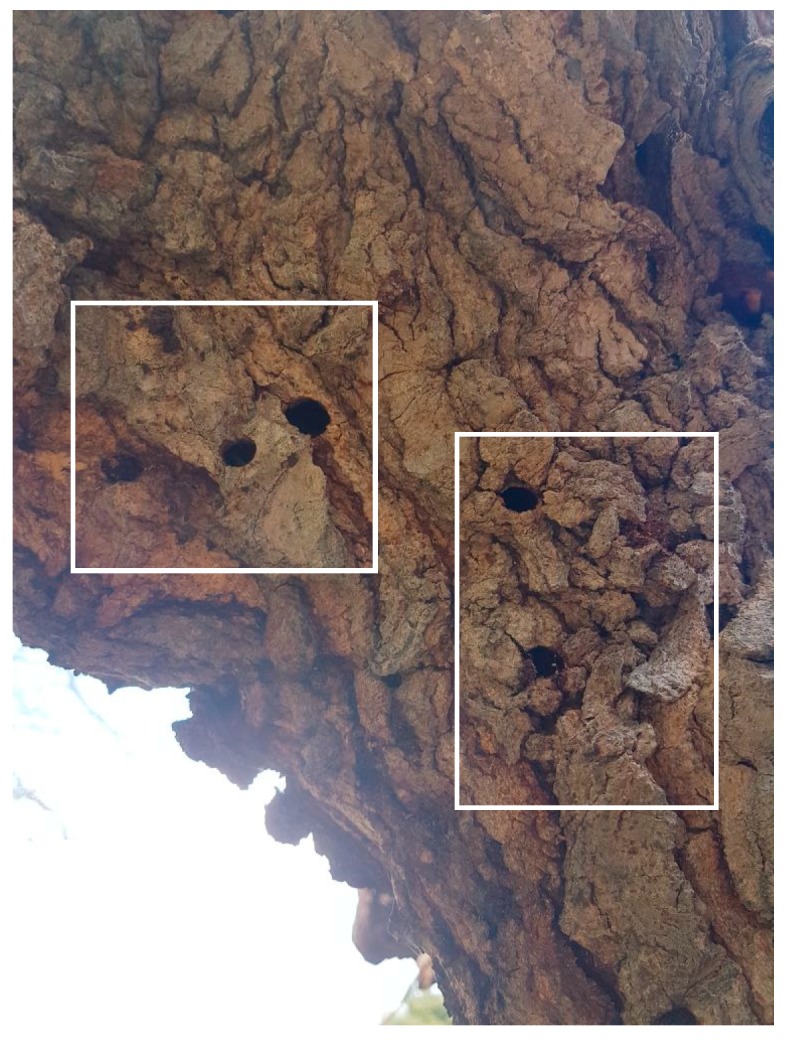
A suspicious tree-trunk: Exit tunnels of adult *X. chinensis* in a mulberry tree marked with a white rectangle. Although the pests have left the tree through the tunnels they have dug, it is mostly probable that there is another generation of larvae breeding that will lead to the next generation of adult insects.

As a means to evaluate performance and in order to confirm a positive prediction of an infestation inside a tree, we need to slice the tree trunks with a chainsaw. This is a laborious process as each tree offers different difficulties during the cutting process (see Figures S1–S3 in the Supplementary Materials). The mulberry is characterized by its solid, hard wood and since the location of the larva or adult insect is unknown, a number of thin slices may be needed whereas palms have a moisty fibrin structure and their juices can corrode the saw in the long run. Healthy trunks have been taken from trees that are not known hosts of the insects investigated, whereas suspicious tree-trunks had marks of exits of adults (see Figure 6) and/or sawdust signs. Note that the exit tunnels indicate that the larvae have become adults that escaped from the tree and there is no guarantee that another generation of larvae is breeding inside. However, a new infestation is usually the case.

**Figure 7:**
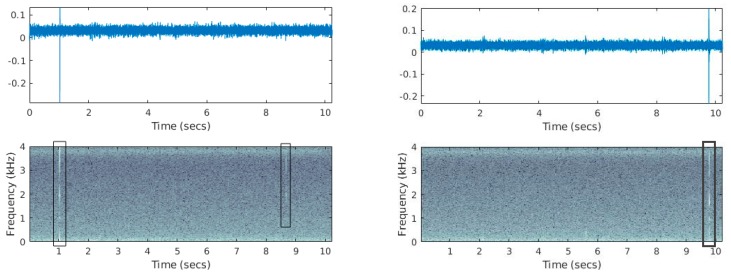
Mulberries positive for *X. chinensis* (trees chopped and alive larvae found). (**top**) time domain recording of the vibrations inside a tree trunk acquired using the suggested device, (**bottom**) Spectrogram of the same recording (i.e., time over frequency representation of the vibroacoustic signal). We mark with rectangles the evidence of audio impulsive sounds from inside the tree shown as white stripes that led into classifying the trunk as infested.

**Figure 8:**
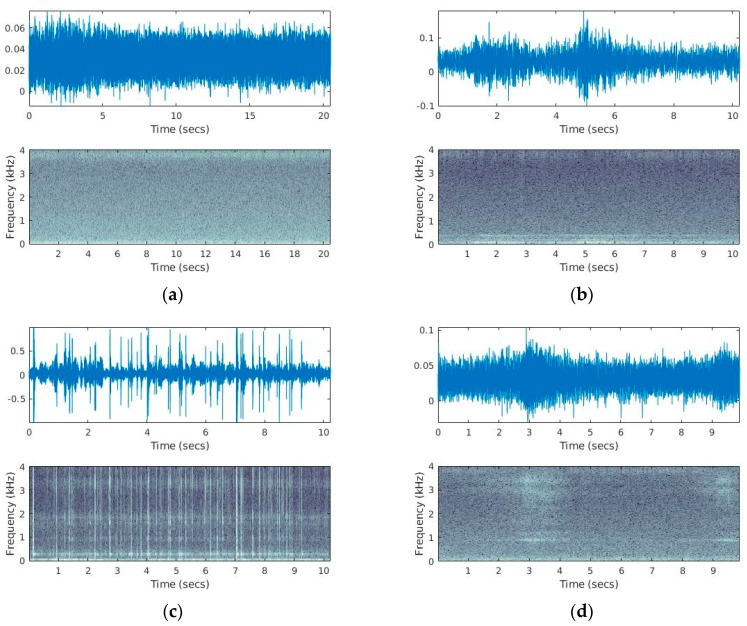
Field-recordings in different mulberries that are negative for the presence of a borer under different environmental conditions. (**a**) A typical case with no signs of any vibration, (**b**) a recording in the presence of strong wind (stormy weather). (**c**) heavy rain hitting the trunk. The impulses are due to the raindrops. (**d**) recordings in the presence of heavy traffic in a rush hour.

Feeding larva produce impulsive audio events that sound like snaps (i.e., breaking fibers) and appear in the spectrogram of vibration recordings as high-energy, broadband stripes (see Figure 7 and listen to Recordings S1–S4 in the Supplementary Materials). Activity rates can vary greatly depending on the infestation level, the biological cycle of the pest and the distance from the larva ranging from few hits every ten seconds to hundreds when near to a highly infested area of a trunk (listen to Recording S1). These sounds are visually and acoustically distinctive when not totally masked by another sound. Although background noise can sometimes be audible, it is heavily attenuated as the waveguide attached to the accelerometer is protected inside the wood. In Figure 8, we demonstrate several negative cases for the presence of borer taken from trees in an urban environment and the impact of background noise. Visual examination of the spectrogram allows to quickly scan a small to medium number of recordings. A spectrogram is a representation based on the short-time Fourier transform that reveals how the energy of the signal is distributed over frequency with respect to time. The recording is chopped to 512 samples chunks with 50% overlap and a Hamming window is applied to each chunk. Each time-domain chunk is Fourier transformed and the log amplitude of all transformed chunks are stacked together to make the time-frequency representation seen if Figure 8. In Figure 8a, we see a typical example of a case where nothing interesting happens. This is a typical case and the figure of a flat spectrogram allows us to discard this recording immediately from further investigation. Figure 8b shows a characteristic recording of the probe inside the tree in the presence of strong wind. In Figure 8c, we can see the vibrations caused by raindrops in the case of heavy rain. In this tree, the smaller branches have been removed and there are no leaves. Therefore, there is a direct path for the raindrops onto the tree trunk. In Figure 8d, the probe is inserted in a tree on the pedestrian side of a road in heavy traffic hours. 

The advantage of our approach is evident as the observer is not obliged to discern in-situ the infestation state of the tree but can decide in the convenience and silence of the laboratory by examining recordings of several days and picking the most high quality recordings taken e.g., the ones during a silent night in order to base ones decision. Screening of recordings must be applied automatically when the number of recordings reaches the range of thousands of cases. Though we feel this is a route that must be investigated independently from this work, we suggest that can be carried out by counting the peaks exceeding a signal-energy threshold either in the time or frequency domain, or semi-automatically by transmitting only the suspicious recordings (i.e., counts exceeding a threshold) to a human observer for further consideration. The latter approach would also result to considerable power saving. In what follows, we present characteristic results from in-lab experiments with healthy and infested tree trunks followed by field applications.

### 3.1. In Lab Experiments

**Figure 9:**
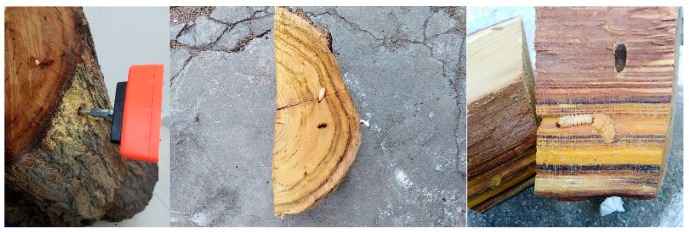
A *X. chinensis* positive case in a mulberry trunk. (**left**) The sensor mounted horizontally in a suspicious trunk in the laboratory, (**middle** and **right**) two alive larvae have been found after cutting the suspicious trunk in slices. The larvae tunnels can be seen in the right picture.

In Figure 9, we show the positioning of the device on a tree trunk. First, we make a hole with a matching drill and we fasten the waveguide tight inside the hole. 

The device registered impulsive sounds and the trunk was classified as infested. In Figure 9-right we see the first live larva of *X. chinensis* we discovered along with two visible tunnels.

### 3.2. Tree Inspections in the Field

We are mostly interested in the open-field case especially in urban environments as we aim at integrating our approach in the context of smart cities and introducing a new surveillance service: remote vibroacoustic surveillance at large spatial scale of trees for borers. We designed a test involving eleven trees in the city of Heraklion in Crete, Greece. The device pinpointed eight trees to be positive for borers and we acquired special permission from the municipality to cut the mulberries down partially (i.e., removal of branches) or totally. Results are gathered at Table 1. 

**Figure 10:**
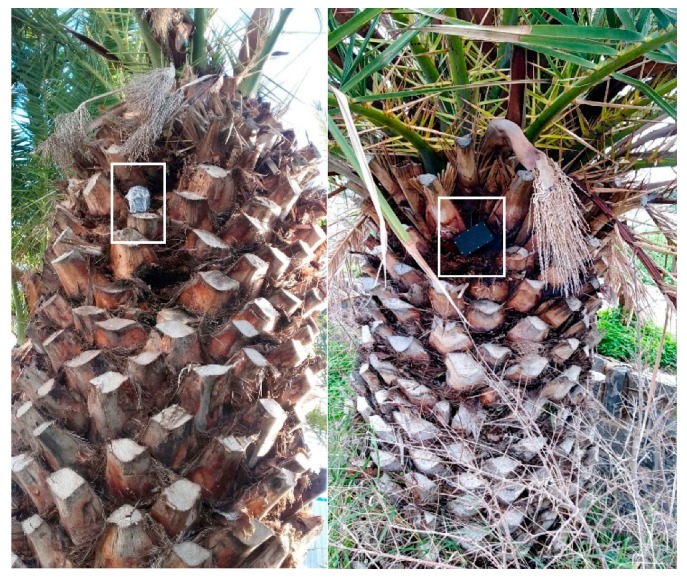
The device attached to a palm tree listening for *R. ferrugineus*. The white rectangle is located around the device.

It is very difficult to recognize the first signs of *R. ferrugineus* presence and only later symptoms are conspicuous. The palm tree at 35°19′37.3′′ N, 25°17′46.3′′ E was selected because it had visible signs of damage on the leaves. The device did not register any impulsive signals. The palm has been inspected manually by checking all leaves for exit tunnels and signs of rotten tissue. Two observation windows were opened by removing four leaves for each window, but no tunnel, larva odor or fungi have been observed. In the case of the palm tree at 35°18′35.26′′ N, 25°06′45.66′′ E there was evidence of yellowing of leaves, fall of lower leaves and crown wilting, therefore the tree was a prominent example of infestation. Again, the device and careful manual inspection have been negative. As the palm was in the secured area of a state’s institution, we asked about the history of the tree and were informed that half a month earlier the tree had been treated with insecticides on the crown but insecticides had also been injected inside the tree from the crown down. These are notable cases because there were visible signs of degradation but the trees were actually healthy. These cases support the argument that the capability to observe remotely and basing the decision on the infestation status after several days of observing the tree’s internal vibrations offers a benefit to visual observations and is useful for after treatment assessment (see Figure 10). 

Solid wood is easier to checked for borers. In the case of mulberries, most cases with background noise due to anthropogenic noise or weather conditions do not result into recordings containing impulsive audio events with the exception of rain. On the other hand, palm trees, due to their fibrin structure can produce easily impulsive events that resemble noises from larvae. In such case, one must avoid making decisions based on recordings that were taken on a windy day and should try to detect pulse trains instead of isolated audio events. Pulse trains are repetitive series of pulses, separated in time and often occur when larvae move or chew. Pulse trains in palms due to wind do not show the systematic nature and compact structure of larvae originating audio events.

In all cases, it was deemed very convenient to listen through the server, one after the other, the trees at different hours and days and switching to listening between adjacent trees in a town square to find out if there is a correlation between detected events indicating that the infestation is spread at least locally. In the case of mulberries, the sensor is placed in the mid-upper part of the trunk, at the base of the main branches joining the trunk. In [10] it was found that this was the most probable location for *X. chinensis*. In the case of palm trees the sensor is placed near the stem or in an open wound as this is the most common entrance point or the red palm weevil. In heavily infested trees the larvae can be anywhere in the tree but for moderate infestations these are the most common locations we find them.

### 3.3. List of Applications

In Table 2, we gather possible applications that can be carried out by the proposed device. Some of them are originating from different domains: 

App #1: We envision a widely applied device (i.e., one for each tree for urban environments) and a sample of trees in forests that allows one to listen to the internal audio scene of a tree in view to detect on time the feeding and locomotion sounds of larvae or adult wood-boring pests digging their exit tunnels.

App #2: We suggest that the same device but with a small bolt or a drill bit functioning as a waveguide can be an integral part of wooden pallets for the transportation of goods. When the cargo would reach the harbor, the pallets would wirelessly transmit their total internal vibrational history to phylosanitary services located in the harbor to be screened automatically before granting free movement of goods. The goal is to intercept wood-infesting insects breeding in pallets (for this application, manually treated, see [23]).

App #3: The suggested device can be used for detection of illegal chopping of trees or unauthorized movement of wooden logs, scented trees etc. The suggested device can be configured to auto-trigger and send an alert e-mail when it receives a shock.

App #4: Tree trunks of palm-trees for transplantation remain in the harbor in a quarantine state for some time. Phylosanitary managers usually inspect them. This service is further facilitated, as one needs a non-specialist only to attach the device on the trunks. The trees will be monitored automatically during their quarantine time. This is especially useful as manual inspection is costly, inconvenient and does not scale to large numbers of applied visits. In addition, the observer may happen to inspect an infested tree at a time when a larva is not active.

App #5: The device can be inserted in the roots of plants in nurseries to detect larvae feeding on the root system of several plants.

App #6: Houses that make extensive use of wood in their construction are popular in the US and in the North of Europe among other places. In the context of smart homes, a device can constantly watch the house in several of its weak spots for the presence of termites and other woodworms. The device alarms the inhabitant by sending an e-mail if the vibration signs originating within the wood persist over time.

App #7: Automated monitoring of stored product pests in silos.

App #8: When inserted in the ground the device can serve as a spatially localized seismometer that can track the passage of heavy vehicles. Therefore, it can be deployed at large numbers for border control of vehicles and is very cost-effective and easily deployed compared to cameras.

## 4. Discussion

Growing a vibrant urban forest requires maintenance. We presented a very efficient automatic recorder for sensing, registering and wirelessly emitting the acoustic emissions that the larvae produce during eating and feeding inside the trunk of a tree. The prototype system is at product level and is portable (half the size of a mobile phone) and was deployed and tested on-site in a real-field setup as well as in the laboratory. Ιt proved to be highly accurate in detecting borers and provided a quick check-up of a large number of mulberry and palm trees with no human intervention which makes it especially suitable for checking out tree trunks in quarantine areas. The device also helps to reduce the cost of treatment and after-treatment analysis of trees using systemic pesticides. After a successful treatment, one should observe a drop of larvae-originated impulsive sounds that must end to a stop in case of a successful treatment. Without an automated procedure, the cost of after treatment monitoring escalates abruptly as the observers need to visit in an iterative mode the same tree to reassess the situation. The novelty of this approach is that the decision can be based on a large time span (e.g., by observing the recordings over a week) and the decision is taken remotely without the need of manual visits. The reason for choosing *X. chinensis* and *R. ferrugineus* was the abundancy of these pests in the island of Crete but the same approach can be applied to different trees and borers around the world by adjusting the length of the waveguide to the targeted trunk e.g., the mountain bark beetle, various longhorn beetles, *Rhynchophorus palmarum* and the *Anoplophora chinensis*.

The proposed solution expands directly to a network of nodes that can cover the trees of a town and which communicate via the low power, LoRa/WAN to a central gateway situated within 1–2 km of the forest plot. This gateway will communicate via satellite or GPRS network to a central server that makes the data available to the end user via a web platform. The LoRa/WAN protocol is optimized for low power consumption and is different to the GPRS we currently use. The first requires that few parameters are transmitted and not complete recordings. The advantage of LoRa is that it can scale to thousands of tree-nodes and fits perfectly in the urban environment. In such case the number of peaks standing out of an energy threshold, the hits rate and the RMS level of the signal can become the communicated data. If one applies two waveguides on a tree then it can record the direction of arrival (DOA) of an audio source. A consistent DOA implies that an audio source is persisting inside the tree whereas random audio events due to tree movement by exogenous audio sources would map to random DOAs. Three waveguides or more on a single tree would allow the localization (i.e., the x-y-z coordinates) of consistent and persisting audio sources based on mapping time delay of arrival (TDOA) to coordinates. In such a case, the manager could even see the tunnels that the larvae dig in the tree. However, to our point of view, more than one waveguide per tree is not currently practical for large-scale deployment.

Our approach fills a need in literature and practice for more spatial data regarding trees and their infestation status with wood-boring insects. The application of our approach will be facilitated when the 5G network is widely accepted and will acquire a completely new meaning when the coverage of internet reaches planet level.

Future work shall focus on: (a) minimizing the cost of the device. Although already cost effective compared to skilled manual operation, there is a large margin of improvement especially on reducing the cost of the piezoelectric probe, (b) further reduction of the size of the device so that it is almost visually imperceptible when installed on a tree and, (c) its integration with energy harvesting methods so that it is permanently attached to a tree and, hopefully, monitors it through its whole life span.

## Figures and Tables

**Table 1:** Field application using the suggested device in the city of Heraklion in Crete. All trees have been partially or totally cut down, sliced and examined. Mulberries and the common fig have been checked for the presence of *X. chinensis* and palm trees for *R. ferrugineus.* ^†±^ two cases correctly classified as negative contradicting visual evidence.

Tree Species	GPS Coordinates	Remote Assessment	Manual Verification
Mulberry	35°19′55.7′′ N, 25°08′03.5′′ E	Positive	Positive
Mulberry	35°19′56.7′′ N, 25°08′04.5′′ E	Positive	Positive
Mulberry	35°19′56.4′′ N, 25°08′04.7′′ E	Positive	Positive
Mulberry	35°19′56.8′′ N, 25°08′05.1′′ E	Positive	Positive
Mulberry	35°20′05.3′′ N, 25°09′50.1′′ E	Positive	Positive
Mulberry	35°20′05.4′′ N, 25°09′50.5′′ E	Positive	Positive
Mulberry	35°20′05.4.8′′ N, 25°09′50.3′′ E	Positive	Positive
^†^Palm tree	35°19′37.3′′ N, 25°17′46.3′′ E	Negative	Negative
^±^Palm tree	35°18′35.26′′ N, 25°06′45.66′′ E	Negative	Negative
Palm tree	35°19′46.3′′ N, 25°12′13.5′′ E	Positive	Positive
Common fig	35°19′09.8′′ N, 25°07′′ 53.2 E	Negative	Negative

**Table 2:** A list of applications directly treated by using the suggested device.

#	Applications
1	Detection of wood-boring insects in urban trees and trees of special/historic interest
2	Self-monitored wooden pallets for transportation of goods
3	Detection of illegal chopping of trees or unauthorized movement/theft of wooden logs
4	Inspection of quarantine trees transported for plantation in harbors
5	Insertion of probe to the ground to detect larvae feeding on the root system of several plants
6	Smart home monitoring of wooden houses and structures for termites
7	Detection of stored product pests in silos
8	Heavy vehicles detection and counting, perimeter security, border control

## Supplementary Materials

The following are available online at https://www.mdpi.com/1424-8220/19/6/1366/s1, Figure S1: a part of a mulberry trunk examined in the lab. Figure S2: Once impulsive sounds are picked up from inside the trunk the validation procedure starts (slicing and examination). Figure S3: Larvae found inside the trunk. Recording S1: xylotrechus_chinensis_piezo_sensor.wav, Adult *X. chinensis* digging their tunnels out in trunk in the laboratory. Vibroacoustic recording from a piezoelectric sensor. Recording S2: xylotrechus_trunk_lab_1.wav, impulsive sounds in trunk in the laboratory. Recording S3: xylotrechus_trunk_lab_2.wav, another example of impulsive sounds in trunk in the laboratory, Recording S4: xylotrechus_tree_field.wav, impulsive sounds in trunk in the field.

## Appendix A

Power consumption depends on user requirements. We provide an example of schedule that records 24 files/day with a duration of 10 s each that are uploaded once per day. The battery capacity is 3000 mAh, the CPU current during sleep mode is 40 uA, and the recording current is 70 mA, the GPS active current: 60 mA and the GSM transmission current (mean value): ~500 mA. The file upload time is 10 s. Therefore:

Recording current (mean value) = (10 s/3600 s) × 70 mA = 194 uA
File upload current (mean value) = ((24 files * 10 s)/86400 s) × 500 mA = 1.38 mA
GPS current (mean value): (240 s/86400 s) × 60 mA = 166 uA
Total Current: 194 uA + 1.38 mA + 40 uA + 166 uA = 1.78 mA
Final battery duration = 3000 mAh/1.78 mA = 1685 h/24 = 70 days

Note that power sufficiency can be extended with the use of a solar panel matching the back of the device.
