# Supplementary figures and images for: In-Vivo Vibroacoustic Surveillance of Trees in the Context of the IoT
Source: Sensors (Basel). 2019 Mar 19;19(6):1366. doi: 10.3390/s19061366 (PMC6471019; doi:10.3390/s19061366)

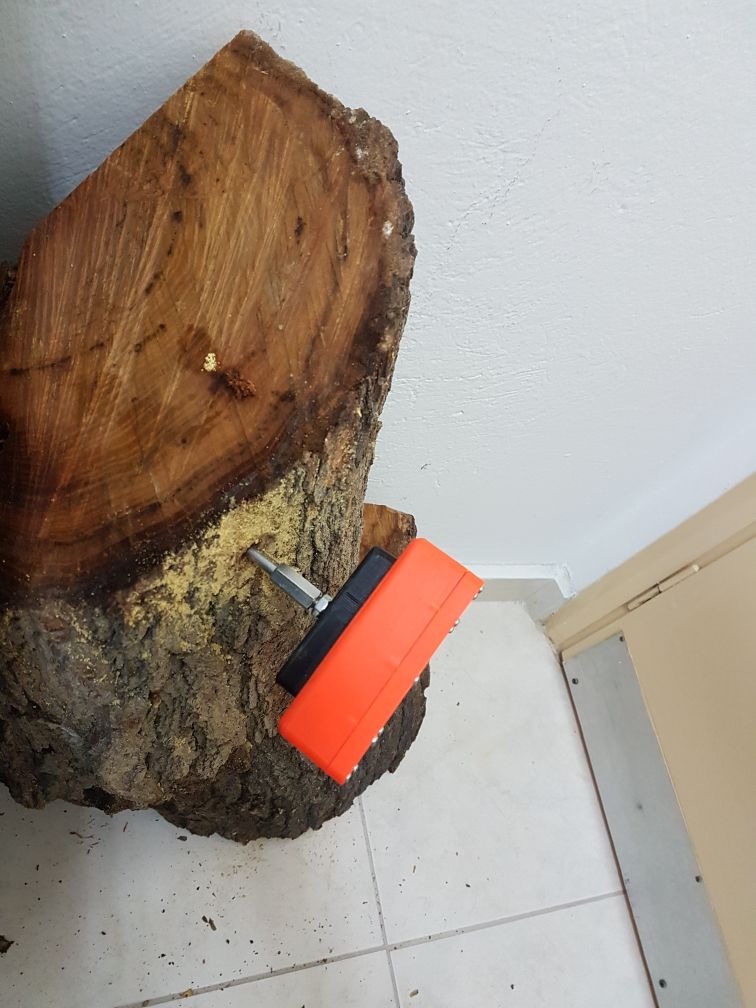

Supplement: Supplementary file 1 [file sensors-19-01366-s001.zip › S1_trunk_in_lab.JPEG]

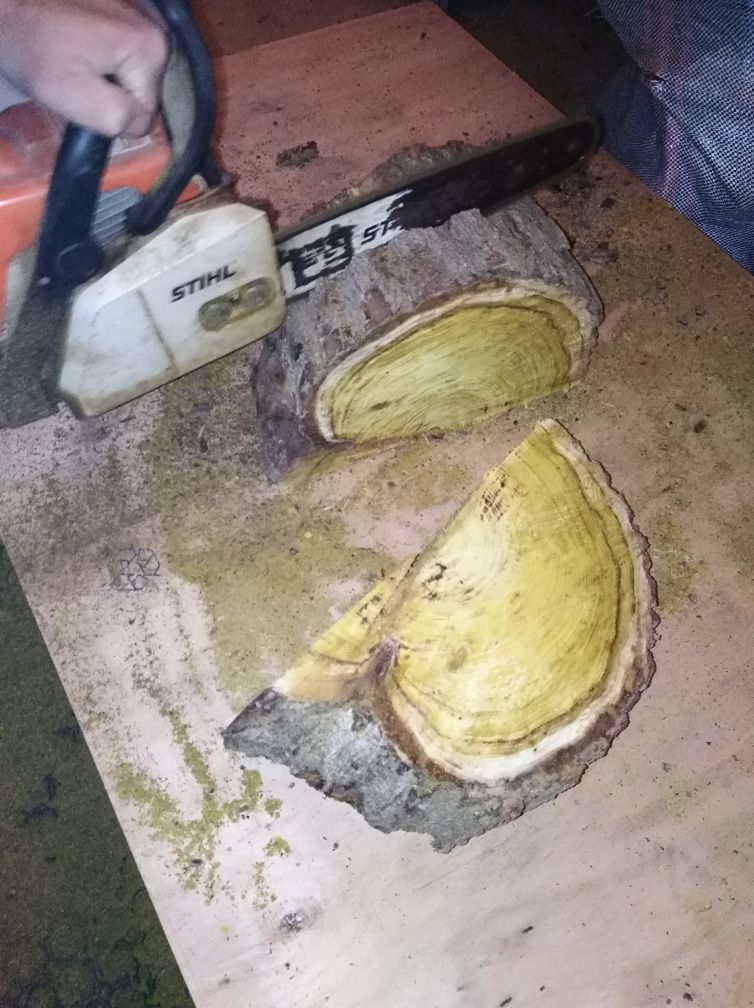

Supplement: Supplementary file 1 [file sensors-19-01366-s001.zip › S2_trunk_validation.JPEG]

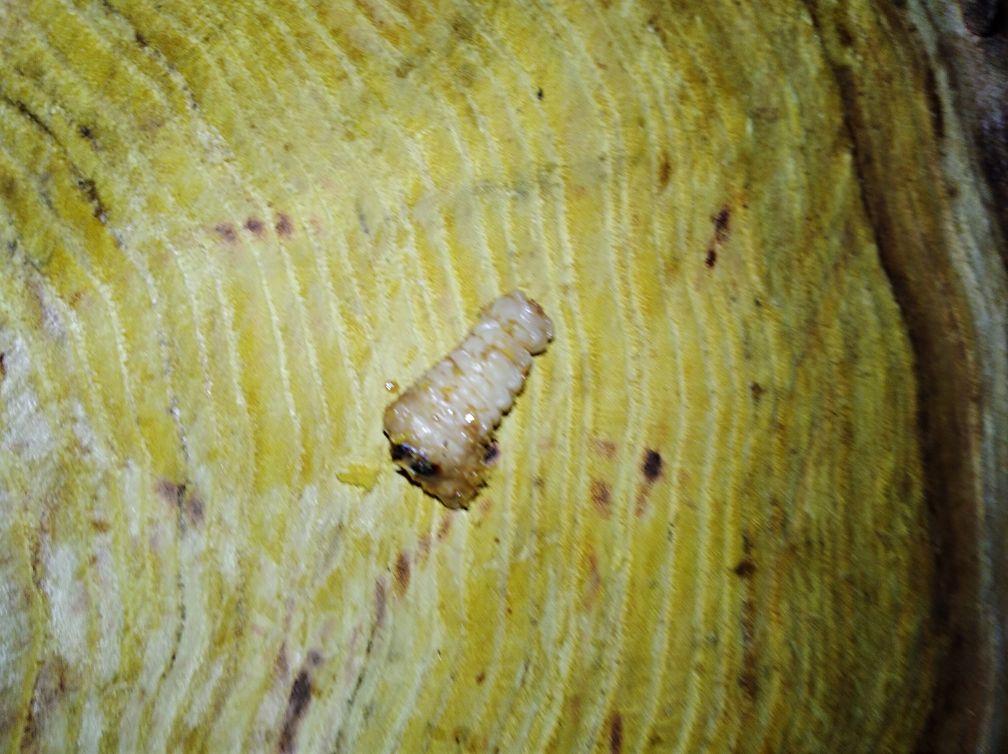

Supplement: Supplementary file 1 [file sensors-19-01366-s001.zip › S3_larva_found.JPEG]
